# A nomogram for predicting postoperative hypernatremia risk in male nonfunctioning pituitary adenomas: A two-center retrospective observational development and validation study

**DOI:** 10.1097/MD.0000000000048154

**Published:** 2026-03-27

**Authors:** Shengwu Lin, Jun Li, Wenxian Yang, Jiansheng Zhong, Xiandong Zeng, Ping Guo, Shousen Wang

**Affiliations:** a Department of Neurosurgery, 900th Hospital of PLA Joint Logistic Support Force, Fuzhou, Fujian, China; b Fujian Provincial Clinical Medical Research Center for Minimally Invasive Diagnosis and Treatment of Neurovascular Diseases, Fuzhou, Fujian, China; c Anhui Provincial Hospital, Bengbu Medical University, Bengbu, Anhui, China; d Fujian Medical University, Fuzhou, Fujian, China; e Department of Neurosurgery, Xiapu County Hospital, Ningde, Fujian, China.

**Keywords:** hypernatremia, nomogram, nonfunctioning pituitary adenomas, predictive model, transsphenoidal surgery

## Abstract

This study aimed to develop and validate a nomogram for predicting postoperative hypernatremia risk in male nonfunctioning pituitary adenoma (NFPA) patients undergoing transsphenoidal surgery. A retrospective analysis was performed on the clinical data of 376 male patients with NFPAs who underwent transsphenoidal surgery at Fuzhou 900 Hospital and Anhui Provincial Hospital between January 2022 and December 2024. Patients from Fuzhou 900 Hospital were divided into the training set (a total of 275 cases, among which 51 cases were hypernatremia) for model development, while patients from Anhui Provincial Hospital were used as the external validation set (a total of 101 cases, among which 17 cases were hypernatremia). Univariate analysis and least absolute shrinkage and selection operator regression were used for preliminary screening of candidate risk factors for postoperative hypernatremia, followed by multivariate logistic regression analysis to identify independent predictors. A predictive nomogram was constructed based on these independent predictors using R software, and the performance of the nomogram was systematically evaluated in 3 domains: discriminative ability, calibration, and clinical utility. Univariate analysis and least absolute shrinkage and selection operator regression identified 7 candidate variables, and multivariate logistic regression confirmed 4 independent predictors of postoperative hypernatremia: preoperative cortisol level (odds ratio [OR] = 0.153, 95% confidence interval [CI]: 0.057–0.406, *P* = .002), intraoperative cerebrospinal fluid leak (OR = 8.40, 95% CI:1.36–51.9, *P* = .022), pituitary stalk morphological change (OR = 6.47, 95% CI: 1.09–38.60, *P* = .040), and pituitary stalk deflection angle difference (OR = 2.31, 95% CI: 1.22–4.40, *P* = .001). The constructed nomogram showed excellent discriminative ability in the training set (area under the receiver operating characteristic curve = 0.8645, 95% CI: 0.7499–0.9791) and good performance in the external validation set (area under the receiver operating characteristic curve = 0.7983, 95% CI: 0.6596–0.9371). Calibration plots and the Hosmer–Lemeshow test (*P* > .05) indicated good calibration, and decision curve analysis and the clinical impact curve confirmed significant clinical utility. A predictive nomogram for postoperative hypernatremia in male NFPA patients was established, integrating preoperative cortisol levels, intraoperative cerebrospinal fluid leak status, and pituitary stalk dynamic imaging parameters to inform evidence-based, personalized perioperative management.

## 1. Introduction

Pituitary adenoma (PA) is one of the most common intracranial benign tumors, accounting for about 10% of all primary intracranial tumors.^[1,2]^ PAs are categorized into functioning and nonfunctioning types based on the secretion of biologically active hormones.^[3]^ Lacking specific endocrine symptoms, nonfunctioning pituitary adenomas (NFPA) are often diagnosed only when the tumor grows large enough to compress the optic chiasm or adjacent structures. Consequently, they frequently present as macroadenomas (1–4 cm) or giant adenomas (≥4 cm).^[4,5]^ Transsphenoidal surgery remains the primary treatment for NFPAs, aiming to achieve maximal tumor resection, relieve mass effect, and preserve pituitary function.^[6]^ Given the tumor’s proximity to the hypothalamic–pituitary axis, surgical manipulation can easily disrupt the synthesis, transport, or release of antidiuretic hormone (ADH). The resulting ADH deficiency and central diabetes insipidus (DI) constitute the core mechanism underlying postoperative hypernatremia.^[7]^ This electrolyte disturbance not only prolongs hospital stays and increases healthcare costs, but also can lead to severe complications, such as altered mental status, seizures, and even mortality.^[8]^ Although postoperative sodium imbalance has garnered research interest,^[9,10]^ given gender differences in water-electrolyte metabolism, a critical gap remains: no dedicated individualized tool is available to accurately predict postoperative hypernatremia risk specifically in male NFPA patients.

The nomogram, a visual predictive model that integrates multiple factors, has demonstrated considerable value in oncology prognosis assessment.^[11,12]^ Therefore, to address this unmet clinical need, this study focused on male patients with NFPA. By systematically analyzing perioperative variables, we aimed to develop, internally and externally validate, a nomogram for predicting postoperative hypernatremia risk. This tool is intended to provide an evidence-based foundation for the early identification of high-risk individuals and the implementation of personalized perioperative interventions.

## 2. Materials and methods

### 2.1. General information

In this two-center retrospective cohort study, we consecutively enrolled 500 male patients who underwent endoscopic transsphenoidal surgery for NFPA at Fuzhou 900 Hospital and Anhui Provincial Hospital between January 2022 and December 2024. The surgeries were conducted by experienced senior neurosurgeons at each center, with standardized transsphenoidal surgical protocols strictly followed to ensure procedural consistency across the 2 institutions. The inclusion criteria were as follows: postoperative immunohistopathological confirmation of NFPA according to the 2017 World Health Organization classification of pituitary tumors, including silent gonadotroph, silent corticotroph, PIT1-lineage adenomas, and null-cell adenomas, without clinical or biochemical evidence of hormone hypersecretion^[13]^; age of ≥18 years; complete perioperative clinical, laboratory, and imaging data; availability of pre- and postoperative 3.0T pituitary thin-slice magnetic resonance imaging (MRI) at each participating center for detailed tumor and pituitary stalk assessment; and adherence to standardized perioperative fluid and electrolyte management protocol, including weight-based maintenance fluids and electrolyte monitoring at least every 8 hours. The exclusion criteria were as follows: history of prior sellar or craniofacial surgery; preoperative or early postoperative radiotherapy or medical therapy; concurrent intracranial or systemic malignancy; missing key variables, especially postoperative serum sodium levels, pituitary stalk parameters, or thyroid–adrenal axis function indicators; and loss to follow-up within 1 week postoperatively. After applying the inclusion criteria, a total of 376 patients were finally included (275 cases from Fuzhou 900 Hospital and 101 cases from Anhui Provincial Hospital).

The study was approved by the clinical medicine ethics committee of Fuzhou 900 Hospital and Anhui Provincial Hospital. Written informed consent was obtained from all patients or their legal guardians.

### 2.2. Outcome

The primary observational endpoint was the occurrence of postoperative hypernatremia. Dynamic changes in serum sodium levels during the postoperative hospital stay were collected from reviewed electronic medical records and follow-up notes. Hypernatremia was defined as a serum sodium concentration of >145 mmol/L on at least 2 consecutive measurements during postoperative hospitalization.^[14]^ Patients were categorized into hypernatremia and non-hypernatremia groups. All clinical data collection and outcome adjudication were finalized in March 2025.

### 2.3. Data collection

The demographics and baseline characteristics were age; tumor characteristics, namely, maximum tumor diameter; macroadenoma (1–4 cm) or giant adenoma (>4 cm) categorized according to diameter^[5]^; modified Knosp grade (grades 0–3A as noninvasive; 3B-4 as invasive)^[15]^; and presence of cystic changes or hemorrhage on imaging. The surgical variables were extent of resection (assessed within 3 months postoperative MRI, that is, gross total resection, subtotal resection, or partial resection^[16]^); and intraoperative cerebrospinal fluid (CSF) leak. Pituitary stalk imaging parameters were measured by 2 independent reviewer teams (each consisting of a neurosurgeon and a neuroradiologist) at the 2 centers, using ImageJ software (National Institutes of Health [NIH], Bethesda) based on preoperative and postoperative (within 72 hours) MRI scans. Assessments included stalk visibility, length, deflection angle, and respective changes in their values. Inter-rater reliability was evaluated using Kappa statistics (Kappa >0.75 indicates excellent agreement). In case of discrepancies between the 2 reviewer teams, a third senior neuroradiologist was consulted to reach a consensus, ensuring the reliability of imaging measurements. For endocrine function, the variables were levels of thyroid-stimulating hormone, free triiodothyronine, free thyroxine (FT4), and cortisol (COR), which were measured within 48 hours before operation and 72 hours after operation. Postoperative DI was defined as postoperative polyuria (>250 mL/h or >4 mL/kg/h for at least 2 consecutive hours) with concurrent dilute urine (specific gravity of <1.005 or urine osmolality < plasma osmolality).^[17]^ The diagnosis required persistence for at least 2 hours and exclusion of other causes (e.g., excessive fluid administration and diuretic use).^[17]^

### 2.4. Statistical analysis

Continuous data conforming to a normal distribution were presented as mean ± standard deviation and were compared using the Student’s *t* test. Data not conforming to a normal distribution were presented as median (interquartile range) and were compared using the Mann–Whitney *U* test. Categorical data are presented as number (percentage) and were compared using the chi-square test, Yates’ corrected chi-square test, or Fisher exact test. Correlation analysis was performed using Pearson’s correlation test for normally distributed data. A two-sided *P* < .05 was considered statistically significant. All statistical analyses were performed using Statistical Package for the Social Sciences (Version 27.0; IBM Corporation, Armonk).

### 2.5. Model development and validation

Variables were initially screened through least absolute shrinkage and selection operator (LASSO) regression to reduce dimensionality and facilitate candidate selection. Relevant variables from this screening were then entered into multivariable logistic regression analysis to facilitate the identification of independent predictors of postoperative hypernatremia. Based on these independent predictors, a nomogram was constructed using R software (R Core Team [R Foundation for Statistical Computing], Vienna, Austria). Internal validation was performed on the training set using bootstrap resampling (1000 repetitions) to assess model overfitting. External validation was conducted on the independent validation set to evaluate the model’s generalizability. Model performance was evaluated in 3 domains for both internal and external cohorts: discrimination, which was assessed by the area under the receiver operating characteristic curve (AUC); calibration, which was assessed by the Hosmer–Lemeshow goodness-of-fit test and calibration plots; and clinical utility, which was assessed using decision curve analysis and clinical impact curve for the quantify the net benefit across a range of risk thresholds.

## 3. Results

### 3.1. Baseline characteristics and univariate analysis

Univariate analysis identified 11 variables that were significantly associated with the development of postoperative hypernatremia (all *P* < .05), including CSF leakage, pituitary stroke/cystic change, postoperative DI, postoperative pituitary stalk morphology, morphological changes of the pituitary stalk, postoperative pituitary stalk length, pituitary stalk length difference, preoperative pituitary stalk deviation angle, pituitary stalk deviation angle difference, preoperative FT4 level, and preoperative COR level. Conversely, no significant correlations were found between postoperative hypernatremia and other variables: maximum tumor diameter, tumor type, modified Knosp grade, extent of resection, preoperative pituitary stalk morphology, postoperative pituitary stalk deviation angle, preoperative and postoperative thyroid-stimulating hormone levels, preoperative and postoperative free triiodothyronine levels, postoperative FT4 level, and postoperative COR level (all *P* *>* .05, Table 1).

**Table 1:** Comparison of clinical characteristics between the hypernatremia group and the non-hypernatremia group.

Factors	Hypernatremia (n = 68)	Non-hypernatremia (n = 308)	*t/x* ^2^	*P*
Age (yr)	50.73 ± 11.72	50.01 ± 9.88	0.472	.638
MTD (mm)	29.17 ± 6.80	28.69 ± 5.28	0.547	.585
Tumor type				
Macroadenomas	55 (80.88%)	274 (88.96%)	3.323	.068
Giant adenomas	13 (19.12%)	34 (11.04%)		
Modified Knosp grade				
0–3A	57 (83.82%)	273 (88.64%)	1.202	.273
3B–4	11 (16.18%)	35 (11.36%)		
Resection extent				
GTR	57 (83.82%)	265 (86.04%)	4.120	.127
STR	7 (10.29%)	14 (4.55%)		
PR	4 (5.88%)	29 (9.42%)		
Cerebrospinal fluid leakage				
Yes	21 (30.88%)	46 (14.94%)	9.673	.002
No	47 (69.12%)	262 (85.06%)		
Pituitary stroke/cystic change				
Yes	18 (26.47%)	127 (41.23%)	5.124	.024
No	50 (73.53%)	181 (58.77%)		
Postoperative diabetes insipidus				
Yes	28 (41.18%)	57 (18.51%)	16.36	<.001
No	40 (58.82%)	251 (81.49%)		
Preoperative pituitary stalk morphology				
Arc	26 (38.24%)	121 (39.29%)	0.026	.872
Straight	42 (61.76%)	187 (60.71%)		
Postoperative pituitary stalk morphology				
Arc	39 (57.35%)	125 (40.58%)	6.369	.012
Straight	29 (42.65%)	183 (59.42%)		
Morphological changes of pituitary stalk				
Yes	25 (36.76%)	57 (18.51%)	10.889	.001
No	43 (63.24%)	251 (81.49%)		
Preoperative pituitary stalk length (mm)	3.09 ± 1.22	3.08 ± 1.36	0.124	.901
Postoperative pituitary stalk length (mm)	4.68 ± 1.47	5.97 ± 1.72	2.683	.008
Pituitary stalk length difference (mm)	1.15 ± 1.20	2.39 ± 1.35	2.752	.006
Preoperative pituitary stalk deviation angle	17.84 ± 6.51	29.14 ± 9.05	3.301	.001
Postoperative pituitary stalk deviation angle	20.37 ± 8.58	30.56 ± 9.81	1.501	.138
Pituitary stalk deviation angle difference	25.05 ± 7.43	12.18 ± 5.44	4.231	<.001
Preoperative TSH (mIU/L)	1.65 ± 0.85	1.55 ± 0.65	1.15	.250
Postoperative TSH (mIU/L)	0.90 ± 0.35	0.85 ± 0.40	0.88	.379
Preoperative FT3 (ng/dL)	4.49 ± 1.45	4.26 ± 0.97	0.845	.400
Postoperative FT3 (ng/dL)	3.44 ± 0.81	3.27 ± 0.82	0.787	.433
Preoperative FT4 (ng/dL)	15.24 ± 5.40	12.53 ± 2.79	3.158	.002
Postoperative FT4 (ng/dL)	15.62 ± 5.05	13.78 ± 4.07	1.710	.090
Preoperative COR (µg/dL)	7.27 ± 4.34	16.95 ± 5.39	3.170	.002
Postoperative COR (µg/dL)	27.30 ± 8.85	26.93 ± 9.94	0.086	.932

*P* < .05 statistically significant.

COR = cortisol, FT3 = free triiodothyronine, FT4 = free thyroxine, GTR = gross total resection, MTD = maximum tumor diameter, PR = partial resection, STR = subtotal resection, TSH = thyroid-stimulating hormone.

### 3.2. Predictor selection and multivariate analysis

**Figure 1.**
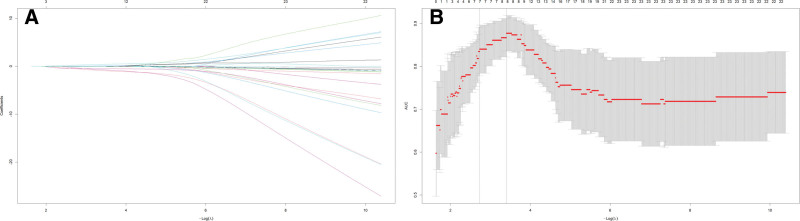
Variables selection using the LASSO logistic regression model. (A) Coefficient paths of the 25 candidate clinical predictors across a range of log(λ) values. (B) Ten-fold cross-validation for tuning parameter (λ) selection. The vertical dashed line indicates the optimal λ value selected according to the minimum criteria (left line) and the 1 standard error of the minimum criteria (right line). The 25 candidate variables included age; maximum tumor diameter; tumor size category; modified Knosp grade; extent of resection; intraoperative cerebrospinal fluid leak; presence of tumor cystic change or hemorrhage; postoperative diabetes insipidus; pre- and postoperative pituitary stalk morphology, length, and deflection angle, along with their dynamic change values; and pre- and postoperative pituitary–thyroid axis function indices (thyroid-stimulating hormone, free triiodothyronine, and free thyroxine) and cortisol levels. AUC = area under the receiver operating characteristic curve, LASSO = least absolute shrinkage and selection operator.

**Figure 2.**
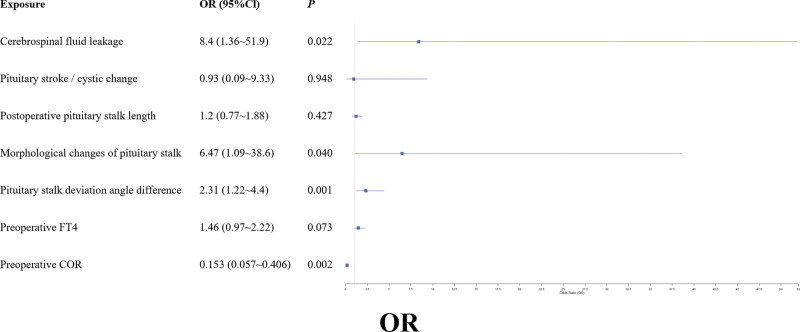
Multivariate logistic regression result graph. OR: odds ratio, also known as the dominance ratio, is an indicator of the strength of the association between disease and exposure in case-control studies. 95% CI: the 95% confidence interval for the OR value, indicating a 95% confidence level that the true OR value will fall within this interval in multiple replicate samples. COR = cortisol, FT4 = free thyroxine.

Using the occurrence of postoperative hypernatremia as the dependent variable, we included 25 candidate predictors in a LASSO regression analysis (Fig. 1A). The optimal penalization parameter lambda (λ = 0.071) was determined through 10-fold cross-validation (Fig. 1B). This process selected 7 variables for further analysis: intraoperative CSF leak, tumor cystic change/hemorrhage, postoperative pituitary stalk length, morphological change of the pituitary stalk, pituitary stalk deflection angle difference, preoperative FT4 level, and preoperative COR level. Subsequently, these variables were entered into a multivariable logistic regression analysis. The results identified 4 independent predictors of postoperative hypernatremia (Fig. 2): preoperative COR (odds ratio [OR] = 0.153, 95% confidence interval [CI]: 0.057–0.406, *P* = .002); CSF leakage (OR = 8.40, 95% CI: 1.36–51.9, *P* = .022); morphological changes of pituitary stalk (OR = 6.47, 95% CI: 1.09–38.60, *P* = .040); and pituitary stalk deviation angle difference (OR = 2.31, 95% CI: 1.22–4.40, *P* = .001).

### 3.3. Construction of the nomogram prediction model

**Figure 3.**
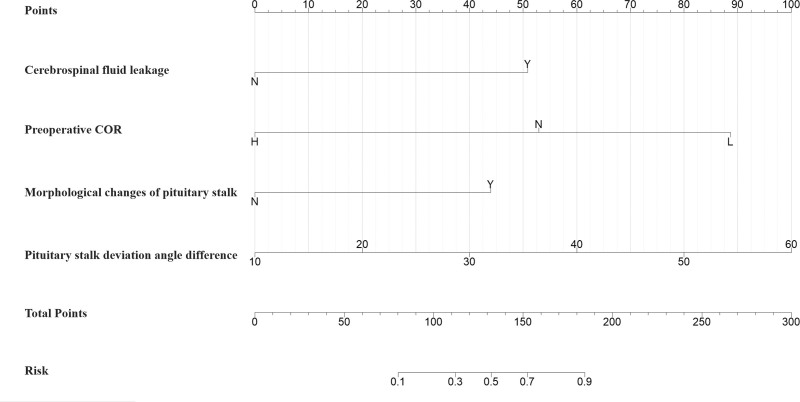
Nomogram incorporating the 4 independent predictors of postoperative hypernatremia (CSF, preoperative COR, pituitary stalk morphological change, and deviation angle difference). The total points calculated from the individual predictors correspond to the predicted probability on the bottom scale. COR = cortisol, CSF = cerebrospinal fluid.

Based on the 4 identified independent predictors, a nomogram was constructed for the individualized prediction of postoperative hypernatremia risk in male patients with NFPA (Fig. 3). This model assigns a specific point value to each level or value of a predictor. The sum of these points corresponds to a predicted probability of risk, as indicated on the bottom scale of the nomogram.

### 3.4. Model validation and performance evaluation

**Figure 4.**
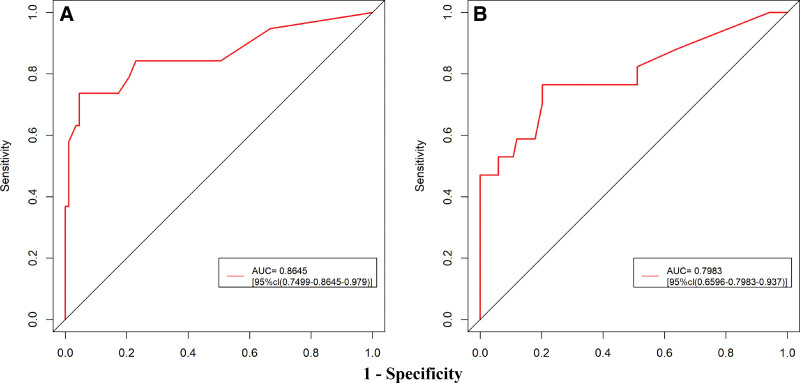
ROC curve of the nomogram for predicting postoperative hypernatremia in male NFPA following transsphenoidal surgery. (A) Development cohort: the red curve represents the ROC curve of the nomogram, with an area under the AUC of 0.8645 (95% CI: 0.7499–0.9791). (B) Validation cohort: the red curve represents the ROC curve of the nomogram, with an AUC of 0.7983 (95% CI: 0.6596–0.9371). AUC = area under the receiver operating characteristic curve, CI = confidence interval, NFPA = nonfunctioning pituitary adenoma, ROC = receiver operating characteristic.

**Figure 5.**
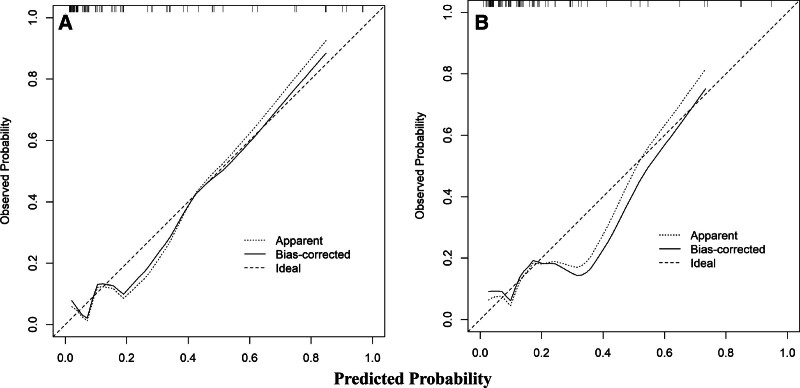
Calibration curve of the nomogram for predicting postoperative hypernatremia in male patients with NFPA. (A) Development cohort: the diagonal dashed line (ideal) represents a perfect prediction where predicted probabilities equal observed probabilities. The solid line (apparent) indicates the apparent predictive performance of the nomogram. The solid line with points (bias-corrected) shows the performance after bias correction through bootstrap resampling (1000 repetitions). Closer proximity of the bias-corrected curve to the ideal line indicates the increased predictive accuracy of the model. (B) Validation cohort: same as (A). NFPA = nonfunctioning pituitary adenoma.

**Figure 6.**
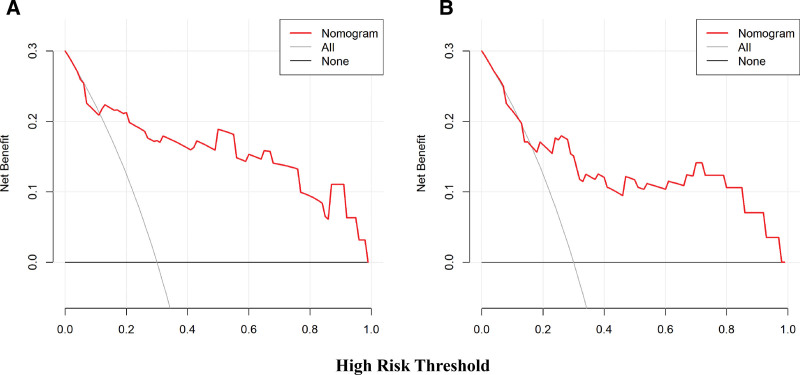
Decision curve analysis of the nomogram for predicting postoperative hypernatremia in male patients with NFPA. (A) Development cohort: the red solid line represents the net benefit of the nomogram across various threshold probabilities. The thin solid line (treat none) indicates the net benefit of the strategy that assumes no patient develops hypernatremia. The thick solid line (treat all) indicates the net benefit of the strategy that assumes all patients develop. (B) Validation cohort: same as (A). NFPA = nonfunctioning pituitary adenoma.

**Figure 7.**
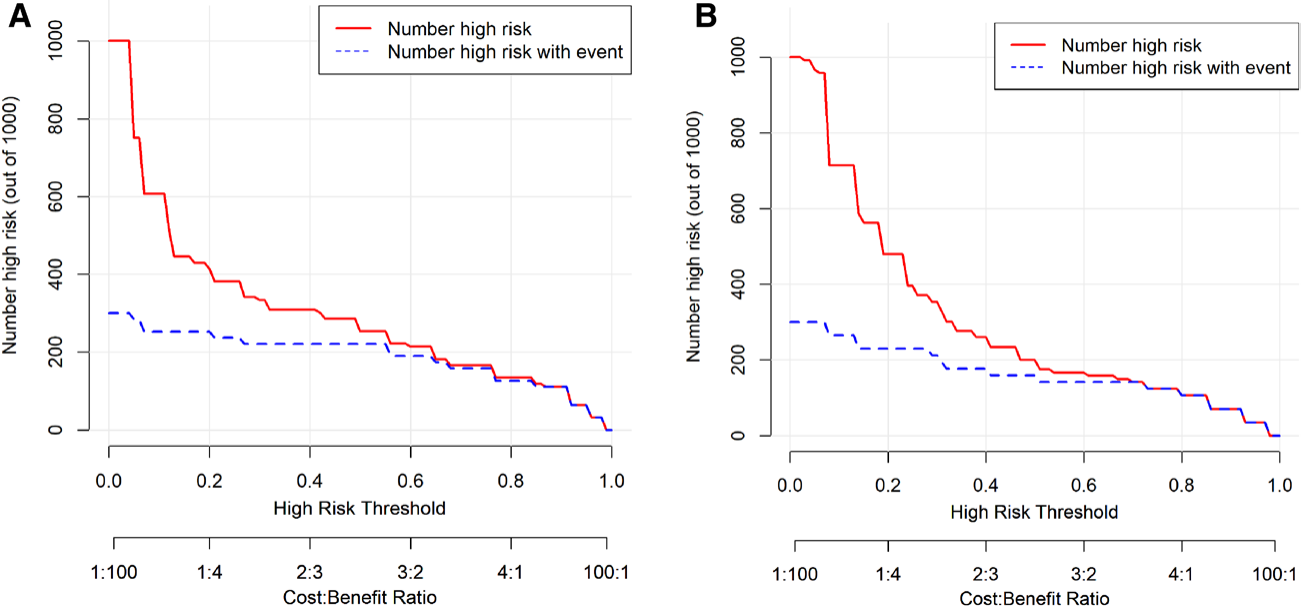
CIC of the nomogram for predicting postoperative hypernatremia in male patients with NFPA. (A) Development cohort: the red solid line represents the number of patients classified as high risk by the nomogram at various risk thresholds. The blue dashed line represents the actual number of patients who developed postoperative hypernatremia among those classified as high risk. (B) Validation cohort: same as (A). CIC = clinical impact curve, NFPA = nonfunctioning pituitary adenoma.

The model’s performance was systematically validated in both the training and external validation sets: internal validation via bootstrap resampling (1000 repetitions) showed excellent discriminative ability with an AUC of 0.8645 (95% CI: 0.7499–0.9791) in the training set (Fig. 4A), while the external validation set maintained good discriminative power (AUC = 0.7983, 95% CI: 0.6596–0.9371; Fig. 4B); calibration plots and Hosmer–Lemeshow tests confirmed good consistency between predicted and observed probabilities in both cohorts (all *P* *>* .05; Fig. 5A and B); decision curve analysis and clinical impact curve further demonstrated substantial net clinical benefit and effective risk stratification across a wide range of clinically relevant thresholds in both sets (Figs. 6A and B, 7A and B), outperforming “treat-all” or “treat-none” strategies.

## 4. Discussion

Postoperative hypernatremia represents a considerable and consequential electrolyte disturbance following transsphenoidal surgery for PAs. Its pathogenesis is closely linked to the injury of the hypothalamic–pituitary stalk region and can lead to severe clinical outcomes.^[18]^ This study is the first to focus on the male NFPA subpopulation. Through a retrospective analysis of 376 patients, we developed and validated a LASSO-logistic regression-based nomogram that successfully identified 4 independent predictors: reduced preoperative COR level, intraoperative CSF leak, preoperative-to-postoperative change in pituitary stalk morphology, and increased difference in pituitary stalk deflection angle. Although postoperative DI was a strong univariate predictor, it was not retained in the final model because it is a secondary manifestation of ADH deficiency rather than a preoperative/intraoperative predictive factor. Our model prioritizes early warning indicators that can be assessed before DI onset, enabling proactive intervention.

As a key stress hormone involved in water and sodium homeostasis, a decreased COR level is a critical indicator of an impaired pituitary–adrenal axis and the risk of secondary adrenal insufficiency.^[19,20]^ This study further supports the conclusions of Bornstein et al^[21]^ and Hannon et al^[22]^ that low preoperative COR levels are associated with the risk of postoperative hypernatremia, while suggesting that it may serve as an independent predictor of this complication. Patients with NFPA may have subclinical adrenal insufficiency preoperatively due to tumor-induced pituitary dysfunction. Surgical trauma, acting as a major stressor, can further challenge this compromised axis and lead to insufficient COR secretion, which impairs renal water reabsorption and exacerbates the risk of hypernatremia. Furthermore, a low COR level may reduce the body’s tolerance to surgical stress and increase the susceptibility of the hypothalamic–pituitary axis to injury. This finding suggests that the preoperative assessment of COR levels and timely glucocorticoid supplementation can mitigate the incidence of hypernatremia.

Our study identified intraoperative CSF leak as an essential predictor of hypernatremia in male patients with NFPA after transsphenoidal surgery. This finding is highly consistent with previous literature identifying CSF leak as a predictor of postoperative DI in patients undergoing endoscopic endonasal transsphenoidal surgery for PAs.^[23–25]^ From an anatomical and surgical perspective, an intraoperative CSF leak directly results from a breach in the sellar floor or diaphragma sellae dura or both, signifying that the natural barrier protecting suprasellar structures (e.g., pituitary stalk and the hypothalamus) is opened. These structures, located in close proximity to or above the diaphragma, become increasingly vulnerable to direct traction, thermal injury, or compression from a sellar packing material when the dura is not intact. Such insults can interfere with their critical functions, including the synthesis and transport of ADH, precipitating DI and ultimately manifesting as hypernatremia.^[26–28]^ Additionally, postoperative management strategies for CSF leak, such as strict bed rest and fluid restriction, may contribute to water-electrolyte imbalance, underscoring the need for intensified monitoring of serum sodium and urine output in these patients.

This study innovatively introduced and confirmed the predictive value of the dynamic imaging changes of the pituitary stalk (including morphological alterations and the increase in the difference in the deflection angle). The pituitary stalk is the sole anatomical conduit for transporting ADH, which is synthesized by hypothalamic magnocellular neurons, to the neurohypophysis.^[29,30]^ Consequently, intraoperative traction, compression, or vascular compromise to this region can disrupt ADH transport.^[31]^ NFPAs, lacking symptoms of hormone hypersecretion, are often diagnosed only after causing mass effects (e.g., visual impairment and headache) and therefore frequently present as macro- or giant adenomas.^[32]^ Such upward-growing tumors commonly displace suprasellar structures, and compression, deviation, or morphological abnormality of the pituitary stalk is a common preoperative MRI finding.^[33]^ After tumor resection, decompression may lead to the elastic retraction of the stalk, or surgical manipulation may cause traction injury, damaging axonal structures and blood supply and resulting in ADH deficiency. The smaller preoperative deflection angle in the hypernatremia group suggests that the stalk may be closer to the midline with tighter anatomical connections to the hypothalamic–pituitary axis, which potentially poses a high intraoperative injury risk. This imaging feature can serve as a valuable preoperative marker for identifying high-risk patients.

A nomogram is a visual mathematical prediction tool that integrates multiple factors by assigning weighted points, enabling individualized outcome probability estimation. It has been widely used for the prediction of recurrence, metastasis, and prognosis.^[34,35]^ However, the application of nomograms for predicting postoperative complications in NFPA is scarce, and specific tools for hypernatremia have not been established. The nomogram developed in this study, based on 4 independent predictors (CSF leak, pituitary stalk morphological change, deflection angle difference, and preoperative COR level), is superior to some existing models in terms of performance. This advantage stems from its precise design for the male NFPA population and the novel incorporation of specific indicators, such as pituitary stalk morphological changes. These metrics capture the aspects of sellar anatomical injury pathways that traditional models undervalue, aligning with the clinical profile of male patients with NFPA, who often have large tumors and pronounced stalk compression. The model has shown good performance in both internal and external validation, which provides a quantitative tool with good generalizability for early identification of high-risk patients and individualized monitoring and intervention.

This study has several limitations. First, although the overall sample size was adequate, the number of hypernatremia events was relatively limited. Future large-scale, prospective, multicenter studies are needed to further validate the model’s stability and generalizability. Second, the model did not incorporate some dynamic physiological parameters (e.g., real-time urine output and serum ADH levels). Future research could explore integrating more real-time monitoring data to enhance predictive performance.

## 5. Conclusion

This study successfully developed and validated a nomogram for predicting postoperative hypernatremia in male patients with NFPAs after transsphenoidal surgery. The model incorporates 4 robust independent predictors: preoperative COR level, intraoperative CSF leak, and pre-to-postoperative changes in pituitary stalk morphology and deflection angle. Demonstrating excellent discriminatory power in internal validation (AUC = 0.8645) and good performance in external validation (AUC = 0.7983), along with satisfactory calibration, this tool provides clinicians with a practical, evidence-based instrument for precise perioperative risk stratification.

## Author contributions

**Conceptualization:** Shengwu Lin, Jun Li, Jiansheng Zhong.

**Methodology:** Shengwu Lin.

**Formal analysis:** Shengwu Lin, Jun Li, Wenxian Yang, Jiansheng Zhong, Xiandong Zeng, Ping Guo.

**Investigation:** Shengwu Lin, Wenxian Yang, Jiansheng Zhong, Xiandong Zeng, Ping Guo.

**Resources:** Shengwu Lin, Wenxian Yang, Jiansheng Zhong, Xiandong Zeng, Ping Guo.

**Software:** Shengwu Lin.

**Validation:** Shengwu Lin, Jun Li, Wenxian Yang, Jiansheng Zhong, Xiandong Zeng, Ping Guo, Shousen Wang.

**Data curation:** Shousen Wang.

**Supervision:** Shousen Wang.

**Writing – original draft:** Shengwu Lin, Jun Li.

**Writing – review & editing:** Shousen Wang.
